# Population-Based Matched Cohort Study of COVID-19 Healthcare Costs, Ontario, Canada

**DOI:** 10.3201/eid3104.241463

**Published:** 2025-04

**Authors:** Beate Sander, Sharmistha Mishra, Sarah Swayze, Yeva Sahakyan, Raquel Duchen, Kieran Quinn, Naveed Janjua, Hind Sbihi, Jeffrey Kwong

**Affiliations:** ICES, Toronto, Ontario, Canada (B. Sander, S. Mishra, S. Swayze, R. Duchen, K. Quinn, J. Kwong); University of Toronto, Toronto (B. Sander, S. Mishra, K. Quinn, J. Kwong); University Health Network, Toronto (B. Sander, Y. Sahakyan); Public Health Ontario, Toronto (B. Sander, J. Kwong); Unity Health Toronto, Toronto (S. Mishra); British Columbia Centre for Disease Control, Vancouver, British Columbia, Canada (N. Janjua, H. Sbihi); University of British Columbia, Vancouver (H. Sbihi)

**Keywords:** COVID-19, viruses, respiratory infections, SARS-CoV-2, healthcare cost, health administrative data, Ontario, Canada

## Abstract

Estimates of COVID-19–related healthcare costs are key to health system planning, but attributable cost data remain limited. We characterized healthcare costs attributable to COVID-19 through a population-based matched cohort study in Ontario, Canada, by using health administrative data. We matched SARS-CoV-2–positive persons from 2020 to unexposed historical control persons from 2016–2018. We estimated phase-based and survival-adjusted COVID-19–attributable healthcare costs from the health system perspective. We matched 159,817 persons. Mean (95% CI) attributable 10-day costs per person were $1 ($–4 to $6) preindex, $240 ($231–$249) during acute care, $18 ($14–$21) in postacute phases, $3,928 ($3,471–$4,384) in the terminal phase for early deaths, and $1,781 ($1,182–$2,380) for late deaths. Mean cumulative survival-adjusted cost at 360 days was $2,553 ($2,348–$2,756) per person. SARS-CoV-2 infection is associated with substantial long-term healthcare costs, consistent with understanding of post-COVID condition. Determining phase-specific costs can inform budget and pandemic planning.

Although only recently emerged, SARS-CoV-2 has negatively affected the health system of Canada (*1*). The health effects of SARS-CoV-2 range from asymptomatic to long-term disability because of the post-COVID condition (PCC), which can lead to substantial long-term costs to health systems (*2*,*3*). PCC is characterized by the continuation or occurrence of new symptoms 3 months after the initial SARS-CoV-2 infection, with symptoms persisting for >2 months without any other explanation (*4*). Surveys report 31% of US adults and 15% of adults from Canada with a history of SARS-CoV-2 infection experienced PCC (*5*,*6*). Emerging data suggest an association between PCC and increased health service utilization for patients requiring both outpatient and inpatient care (*3*,*7*–*9*). Over a 7-month period, healthcare costs nearly doubled for patients with PCC compared with patients without PCC, and anticipated cost increased with extended follow-up (*10*). Because of the high prevalence of SARS-CoV-2 infection (≈83% of adults in Ontario, Canada), evidence of long-term effects of COVID-19, and the effect on health services utilization (*11*), health system coordinators must understand the costs of caring for patients with PCC at different stages of disease and across different sectors of the health system for effective resource allocation and planning.

Previous studies on COVID-19–related costs largely focused on inpatient costs (*12*), the acute period (*13*), or the postacute period (*14*,*15*), potentially underestimating the overall financial effect of COVID-19. The importance of studying longer postinfection periods is evident in recent studies documenting the long-term health (*2*,*3*) and resource use (*7*,*8*) effects of PCC. Our aim was to characterize acute and long-term COVID-19–attributable healthcare costs from the Ontario health system perspective from persons who tested positive for SARS-CoV-2 compared with matched unexposed persons.

## Methods

### Study Design, Setting, and Population

Our study followed the RECORD statement for observational studies (*16*). We conducted an incidence-based matched cohort study to measure the COVID-19–attributable healthcare costs among persons registered in the publicly funded Ontario Health Insurance Plan (OHIP). Ontario is Canada’s most populous province, containing 40% of Canada’s population, and OHIP covers most residents. We used population-based health administrative datasets with information on all healthcare encounters covered by OHIP. We linked the datasets (Appendix Table 1) by using unique encoded identifiers and analyzed them at ICES (Toronto, ON, Canada).

We identified persons as exposed if they tested positive for SARS-CoV-2 by PCR during January 1, 2020–December 31, 2020, enabling 1 year of follow-up, and limiting potential biases because of variant emergence and vaccination beginning in December 2020 (*17*). We defined the index date as the first occurrence of a positive SARS-CoV-2 PCR test on the basis of the earliest of symptom onset date recorded in Ontario’s Case and Contact Management (CCM) system, specimen collection date, observation date or reporting date in the Ontario Laboratories Information System (OLIS), or case report date in CCM. We excluded patients with hospital-acquired SARS-CoV-2 infection, defined as testing positive >8 days after admission or up to 2 days after discharge, given a length of stay of >8 days, or readmission with a positive test within 8 days after discharge from the hospital (*18*–*20*).

We identified unexposed persons by using a 50% random sample of the OHIP-registered population from January 1, 2016–December 31, 2018. We chose a historical unexposed cohort because health service use declined by 27%–43% during the pandemic compared with prepandemic years because of system, structural, and behavioral factors (*21*–*23*) and to avoid potential contamination bias because not all persons with SARS-CoV-2 infection had a PCR test for a variety of reasons, including being ineligible for PCR testing, lacking access to or desire for testing, or being asymptomatic, which ranged from 20%–62% of persons (*22*,*24*; P. Jha, unpub. data, https://europepmc.org/article/PPR/PPR293015). Unexposed persons were assigned a pseudo–index date at random. We excluded persons with death dates before their pseudo–index dates from the study and excluded persons assigned to the historical unexposed cohort from the exposed cohort.

Exclusion criteria for both groups included not residing in Ontario; having invalid or missing gender, birthdate, or income quintile information; being >65 years of age and not having any healthcare system interactions for 3 years before the study start date (January 1, 2020, for the exposed group; January 1, 2016, for the unexposed group); being <65 year of age and not having any healthcare system interactions for 10 years before the study start date; being >110 years of age; and residing in long-term care. We followed all persons for 1 year from their index date.

We used hard and propensity score matching on selected baseline covariates. We matched each exposed person to 1 unexposed person by using nearest-neighbor matching without replacement on index date (day and month) +60 days, sex, age category, resource utilization band with a 2-year look-back window, and the logit of the propensity score by using a caliper distance of 0.2 SD (*25**,**26*). The resource utilization bands were derived from the Johns Hopkins adjusted clinical groups (ACG) and categorized comorbidity into 6 groupings from nonutilizer to high complexity of illness (*27**,**28*).

The propensity score included the following baseline measures: public health unit (PHU) of residence, rural or urban residence, census tract of residence (that are not contiguous with the boundaries of local PHUs), frailty, high-risk occupation neighborhood concentration quintile, immigrant status, and Ontario Marginalization Index (*29*) quintiles for age and labor force, material resources, racialized and newcomer populations, and households and dwellings. We defined frailty by using ACG (*27*). High-risk occupation neighborhood concentration quintile was based on the proportion of residents in a census dissemination area employed as essential workers.

Because healthcare utilization and associated costs tend to increase before death (*30*), we examined attributable cost before death by rematching exposed persons who died to unexposed persons who also died during the observation period on their death dates (day and month) +30 days, sex, income quintile, recent immigrant, and the logit of the propensity score by using a caliper distance of 0.2 SD (*25*,*26*). The propensity score included the following baseline measures: PHU of residence, rural/urban residence, census tract of residence, high-risk occupation neighborhood concentration quintile, and Ontario Marginalization Index quintiles (*29*) for age and labor force, material resources, racialized and newcomer populations, and households and dwellings. We assessed the balance for all matches by using standardized differences with a threshold of 0.10 (*25*,*26*).

### Outcomes

We calculated healthcare costs adjusted for survival over a 1-year period after the index date. Costs in 2023 Canadian dollars were calculated from the Ontario health system perspective and included all publicly funded health services: inpatient hospitalizations, outpatient hospital visits (same-day surgeries, outpatient cancer therapies, and dialyses), emergency department visits, publicly funded drugs (for everyone >65 years of age and select younger persons based on means), physician services, rehabilitation services, complex care, homecare, long-term care, and other (e.g., laboratory tests and services, OHIP-covered nonphysician services, assistive devices).

### Analyses

To calculate survival-adjusted costs, we first estimated healthcare costs, standardized to 10 days, from index date to the end of follow-up or death, by using ICES person-level cost methods (*31*). Next, we used the phase-of-care cost approach to assign 10-day costs to phases of care along the natural history of disease trajectory (*32*). Phase-of-care lengths were determined on the basis of clinician expertise, World Health Organization’s definition of PCC (*4*), and joinpoint analysis, identifying significant changes in the trend of mean 10-day costs (*33*). We defined 4 phases of care: prediagnosis (30 days before index date), acute care (80 days after index date), postacute care (time between acute care phase and terminal phase follow-up), and terminal (60 days before death date). We first defined the terminal phase length, followed by acute, postacute, and prediagnosis phases. To determine the terminal phase length, we analyzed 10-day cost trajectories up to 90 days before death among decedents. Joinpoint analysis identified an inflection point at 60 days before death, marking the start of the terminal phase. We further stratified deaths as early if death occurred within 60 days of index date and late if death occurred >60 days following index date. To determine the acute phase length, we analyzed 10-day cost trajectories after the index date up to 360 days, censoring patients 60 days before death. Guided by the World Health Organization’s definition of PCC, which refers to the continuation or occurrence of new symptoms 3 months after the initial SARS-CoV-2 infection (*4*), and on the basis of joinpoint regression results, we selected 80 days postindex as the acute-phase endpoint. We allocated the remaining costs between the end of the acute phase to end of follow-up or to the start of the terminal phase, whichever occurred first, to the postacute phase. Finally, we analyzed prediagnosis costs up to 360 days before the index date, remaining flat during that period. Guided by expert opinion, we chose 30 days before index as the prediagnosis phase start. We calculated mean attributable phase-of-care-specific costs (standardized to 10 days) and 95% CIs by using a generalized estimating equation model. Last, we combined attributable phase-of-care-specific costs with crude survival data to determine 1-year costs adjusted for survival, as described previously (*32*).

We stratified analyses by age, sex, income quintile, and resource utilization band quintile. We conducted sensitivity analyses and varied the lengths of the prediagnosis (120 days before index date), acute care (30 days post index date), and postacute care (time between acute care phase and terminal phase follow-up) phases. We used SAS Enterprise Guide 7.15 (SAS Institute, https://www.sas.com) for all statistical analyses.

## Results

### Study Cohort

During January 1, 2020–December 31, 2020, in Ontario, 181,979 residents tested positive for SARS-CoV-2 (Appendix Figure 1). Among them, 165,838 met eligibility criteria for the exposed cohort; mean age was 40.4 ± 19.7 years, 50.7% were female, and 49.3% were male. There were 6,641,074 unexposed persons residing in Ontario during January 1, 2016–December 31, of whom 2,018 were eligible for matching; mean age was 40.5 ± 22.8 years, 50.6% were female, and 49.4% were male (Table 1).

**Table 1:** Demographics of persons exposed to COVID-19 and unexposed persons before and after matching, Ontario, Canada*

Characteristics	Prematched				Matched				Unmatched exposed, n = 6,021
	Exposed, n = 165,838	Unexposed, n = 6,641,074	SMD		Exposed, n = 159,817	Unexposed, n = 159,817	SMD		
Age at index, y, mean ± SD	40.4 ± 19.7	40.5 ± 22.8	0.01		40.4 ± 19.8	40.5 ± 19.9	0.01		39.9 ± 18.2
Female	84,013 (50.7)	3,362,838 (50.6)	0.00		80,987 (50.7)	80,987 (50.7)	0.00		3,026 (50.3)
Male	81,825 (49.3)	3,278,236 (49.4)	0.00		78,830 (49.3)	78,830 (49.3)	0.00		2,995 (49.7)
Rural	5,691 (3.4)	95,577 (10.5)	0.28		5,641 (3.5)	5,655 (3.5)	0.00		50 (0.8)
Immigrant	63,962 (38.6)	1,167,364 (17.6)	0.48		60,357 (37.8)	59,435 (37.2)	0.01		3,605 (59.9)
Frail	4,152 (2.5)	124,329 (1.9)	0.04		3,729 (2.3)	5,580 (3.5)	0.07		423 (7.0)
ACG, mean ± SD	4.9 ± 3.5	4.1 ± 3.3	0.24		4.9 ± 3.5	4.7 ± 3.4	0.06		5.4 ± 3.4
Died during follow-up†	3,357 (2.0)	214,864 (3.2)	0.08		3,222 (2.0)	4,437 (2.8)	0.05		135 (2.2)
Neighborhood income, lowest to highest									
1st quintile	40,878 (24.6)	1,298,211 (19.5)	0.12		39,182 (24.5)	39,182 (24.5)	0.00		1,696 (28.2)
2nd quintile	36,212 (21.8)	1,303,219 (19.6)	0.05		34,653 (21.7)	34,653 (21.7)	0.00		1,559 (25.9)
3rd quintile	36,205 (21.8)	1,331,629 (20.1)	0.04		34,731 (21.7)	34,731 (21.7)	0.00		1,474 (24.5)
4th quintile	29,252 (17.6)	1,342,461 (20.2)	0.07		28,385 (17.8)	28,385 (17.8)	0.00		867 (14.4)
5th quintile	23,291 (14.0)	1,365,554 (20.6)	0.17		22,866 (14.3)	22,866 (14.3)	0.00		425 (7.1)
Age and labor force, lowest to highest									
1st quintile	59,199 (35.7)	1,798,679 (27.1)	0.19		57,196 (35.8)	56,584 (35.4)	0.01		2,017 (33.5)
2nd quintile	37,644 (22.7)	1,351,752 (20.4)	0.06		36,069 (22.6)	36,258 (22.7)	0.00		1,582 (26.3)
3rd quintile	26,766 (16.1)	1,163,844 (17.5)	0.04		25,800 (16.1)	25,945 (16.2)	0.00		954 (15.8)
4th quintile	22,439 (13.5)	1,114,963 (16.8)	0.09		21,664 (13.6)	21,702 (13.6)	0.00		754 (12.5)
5th quintile	18,896 (11.4)	1,166,635 (17.6)	0.18		18,245 (11.4)	18,884 (11.8)	0.01		667 (11.1)
Material resource, lowest to highest									
1st quintile	27,993 (16.9)	1,511,973 (22.8)	0.15		27,408 (17.1)	27,528 (17.2)	0.00		594 (9.9)
2nd quintile	28,849 (17.4)	1,390,533 (20.9)	0.09		27,953 (17.5)	28,033 (17.5)	0.00		836 (13.9)
3rd quintile	32,509 (19.6)	1,258,511 (19.0)	0.02		31,358 (19.6)	31,305 (19.6)	0.00		1,215 (20.2)
4th quintile	34,440 (20.8)	1,204,487 (18.1)	0.07		32,887 (20.6)	33,218 (20.8)	0.01		1,558 (25.9)
5th quintile	41,153 (24.8)	1,230,369 (18.5)	0.15		39,368 (24.6)	39,289 (24.6)	0.00		1,771 (29.4)
Racialized and newcomer populations, lowest to highest									
1st quintile	9,144 (5.5)	1,043,527 (15.7)	0.34		8,949 (5.6)	9,544 (6.0)	0.02		147 (2.4)
2nd quintile	13,599 (8.2)	1,100,621 (16.6)	0.26		13,348 (8.4)	13,428 (8.4)	0.00		314 (5.2)
3rd quintile	20,746 (12.5)	1,185,255 (17.8)	0.15		20,191 (12.6)	20,517 (12.8)	0.01		513 (8.5)
4th quintile	35,081 (21.2)	1,395,258 (21.0)	0.00		34,095 (21.3)	34,234 (21.4)	0.00		1,023 (17.0)
5th quintile	86,374 (52.1)	1,871,212 (28.2)	0.50		82,391 (51.6)	81,650 (51.1)	0.01		3,977 (66.1)
Households and dwellings, lowest to highest									
1st quintile	47,713 (28.8)	1,466,510 (22.1)	0.15		45,676 (28.6)	44,950 (28.1)	0.01		2,034 (33.8)
2nd quintile	27,501 (16.6)	1,262,030 (19.0)	0.06		26,486 (16.6)	26,535 (16.6)	0.00		991 (16.5)
3rd quintile	25,258 (15.2)	1,200,446 (18.1)	0.08		24,368 (15.2)	24,579 (15.4)	0.00		903 (15.0)
4th quintile	25,957 (15.7)	1,186,139 (17.9)	0.06		25,084 (15.7)	25,702 (16.1)	0.01		886 (14.7)
5th quintile	38,515 (23.2)	1,480,748 (22.3)	0.02		37,360 (23.4)	37,607 (23.5)	0.00		1,160 (19.3)
Essential worker, lowest to highest									
1st quintile	26,206 (15.8)	1,412,108 (21.3)	0.14		25,780 (16.1)	26,293 (16.5)	0.01		426 (7.1)
2nd quintile	34,294 (20.7)	1,473,288 (22.2)	0.04		33,431 (20.9)	33,151 (20.7)	0.00		863 (14.3)
3rd quintile	32,254 (19.4)	1,312,630 (19.8)	0.01		31,060 (19.4)	31,160 (19.5)	0.00		1,194 (19.8)
4th quintile	35,587 (21.5)	1,267,957 (19.1)	0.06		33,991 (21.3)	33,716 (21.1)	0.00		1,596 (26.5)
5th quintile	37,307 (22.5)	1,162,361 (17.5)	0.13		35,376 (22.1)	35,221 (22.0)	0.00		1,931 (32.1)
Resource utilization band									
Nonusers	12,610 (7.6)	798,484 (12.0)	0.15		12,292 (7.7)	12,292 (7.7)	0.00		318 (5.3)
Healthy users	8,344 (5.0)	412,761 (6.2)	0.05		8,138 (5.1)	8,138 (5.1)	0.00		206 (3.4)
Low RU	29,017 (17.5)	1,327,448 (20.0)	0.06		28,132 (17.6)	28,132 (17.6)	0.00		885 (14.7)
Moderate RU	81,334 (49.0)	2,969,570 (44.7)	0.09		78,153 (48.9)	78,153 (48.9)	0.00		3,181 (52.8)
High RU	25,032 (15.1)	823,659 (12.4)	0.08		24,031 (15.0)	24,031 (15.0)	0.00		1,001 (16.6)
Very high RU	9,501 (5.7)	309,152 (4.7)	0.05		9,071 (5.7)	9,071 (5.7)	0.00		430 (7.1)

*Values are no. (%) except as indicated. ACG, adjusted clinical groups; FU, follow up; n, number of observations; RU, resource utilization; SMD, standardized difference in means. †Deaths that occurred during the follow up until March 1, 2022.

Most exposed persons (96.0%) were matched to an unexposed person. The matched cohort consisted of 319,634 persons (159,817 exposed, 159,817 unexposed); mean age was 40.4 ± 19.8 years, 50.7% were female, 49.3% were male, 24.5% lived in neighborhoods with the lowest income levels, 20.7% had high or very high resource utilization, and 2.3% were frail (mean ACG score 4.9) (Table 1). The matched cohort was well balanced, with no standardized differences above 0.1. Unmatched exposed persons were more likely to live in lower income neighborhoods, were more likely to live in urban areas, had a higher mean ACG score, and were more likely to be frail compared with matched exposed persons (Table 1).

We identified 3,357 exposed persons in the terminal phase and matched 93.0% (n = 3,114) to achieve balanced matches (Appendix Table 2). Within 14 days of the index date, 5.1% of the matched exposed cohort were hospitalized, and 26.5% of those were admitted to an intensive care unit (ICU). During the follow-up, 2% of the matched cohort died, including 20.1% of those who were hospitalized within 14 days of index date without ICU admission and 39.1% of those admitted to an ICU.

### COVID-19–Attributable Healthcare Costs

Mean (median) time contributed by matched exposed persons were 30 (30) days for preindex, 77 (79) days for acute, 274 (280) days for postacute, and 35 (30) days for terminal phases. In the preindex phase, the mean (95% CI) 10-day healthcare costs were similar at $107 ($103–$110) for exposed and $106 ($103–$109) for unexposed persons (Appendix Table 3). 

**Figure 1:**
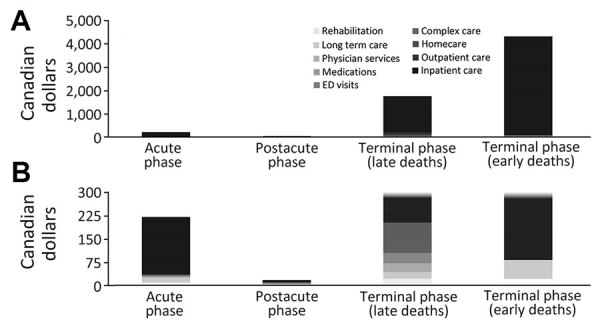
Source of COVID-19–attributable healthcare costs (2023 Canadian dollars), standardized to 10 days by phases-of-care, Ontario, Canada. Cost categories are displayed by phase of care. A) Full data; B) data truncated at $300 to improve visualization of the postacute-phase costs. ED, emergency department

During the acute phase, the mean (95% CI) 10-day health costs were $334 ($325–$343) per person for exposed and $94 ($92–$97) for unexposed persons, resulting in $240 ($231–$249) of COVID-19–attributable costs. Most acute phase costs were because of inpatient care (77%) (Figure 1; Appendix Table 3).

During the postacute phase, the mean (95% CI) 10-day health costs were $112 ($109–$115) per person for exposed and $95 ($93–$97) for unexposed persons, resulting in $18 ($14–$21) of COVID-19-attributable costs. Most postacute phase costs were because of inpatient care (42%) (Figure 1; Appendix Table 3).

During the terminal phase, among persons with early deaths, the mean (95% CI) 10-day health costs were $8,724 ($8,328–$9,119) per person for exposed and $4,796 ($4,551–$5,041) for unexposed persons, resulting in $3,928 ($3,471–$4,384) of COVID-19–attributable costs. Among persons with late deaths, the mean (95% CI) 10-day health costs were $6,709 ($6,194–$7,224) per person for exposed and $4,928 ($4,603–$5,253) for unexposed persons, resulting in $1,781 ($1,182–$2,380) of COVID-19-attributable costs. Most terminal phase costs were because of inpatient care for both early (100%) and late deaths (87%) (Figure 1; Appendix Table 3).

Mean (95% CI) attributable 10-day COVID-19 costs were lower for female than male patients for the acute-care phase, $193 ($182–$204) versus $289 ($274–$304), but higher for female than male patients in the postacute care phase, $21 ($16–$26) versus $14 ($9–$19). Total mean attributable costs in both acute and postacute care phases were higher among older age groups, among those in the lower income quintiles, and in the 2 highest resource utilization bands (Appendix Tables 4–7).

**Figure 2:**
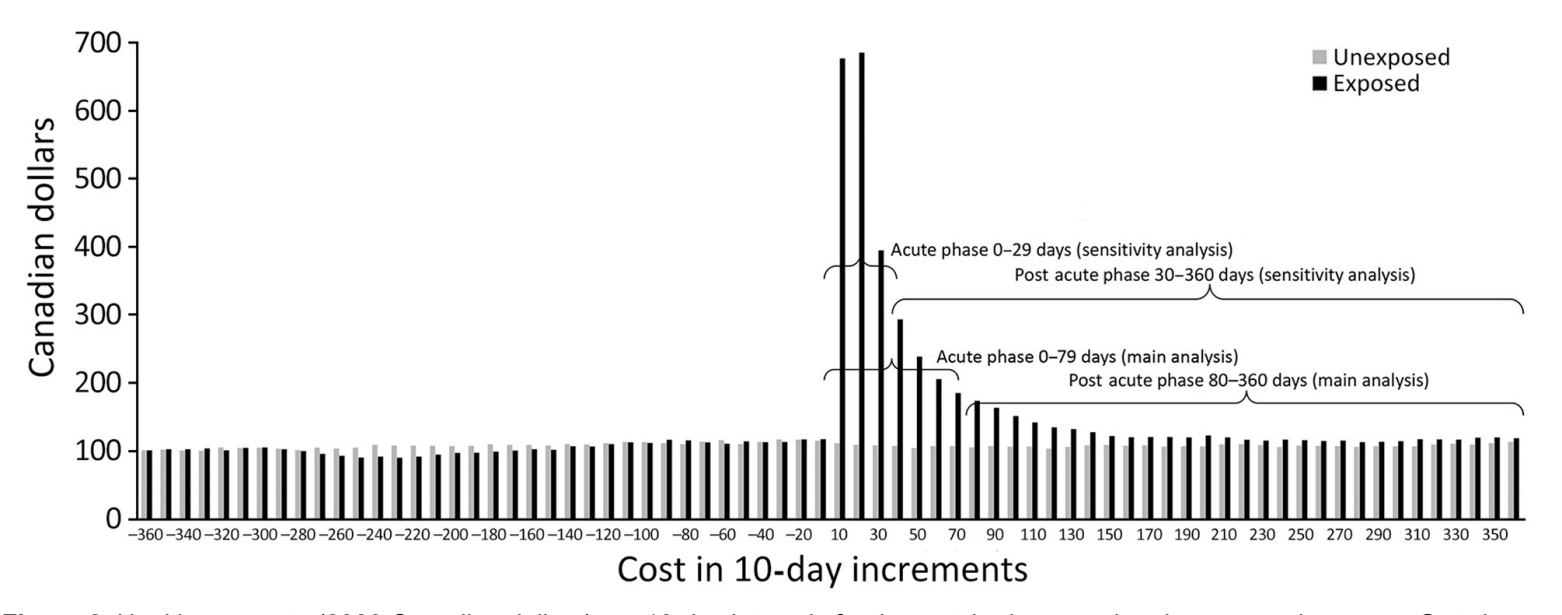
Healthcare costs (2023 Canadian dollars) per 10-day intervals for the matched exposed and unexposed persons, Ontario, Canada. Costs of persons who died during the follow-up period were removed 60 days before death, consistent with the terminal phase length for late deaths.

Healthcare costs were not sensitive to the preindex phase length. However, compared with the phase lengths in the main analysis, costs doubled for the acute and postacute phases when we considered shorter duration (30 days) for acute phase, with inpatient care remaining the main cost driver (Appendix Table 8). This change occurred because costs initially captured under the acute phase were now shifted to the postacute phase (Figure 2). Finally, mean cumulative costs adjusted for survival at 360 days were $2,553 ($2,348–$2,756); costs were lower for female patients, at $2,194 ($1,945–$2,446), versus male patients, at $2,921 ($2,602–$3,241) (Table 2). We summarized healthcare costs of persons who were hospitalized within 14 days of the index date with or without ICU admission (Appendix Table 9).

**Table 2:** COVID-19–attributable costs adjusted for survival, total and stratified by age and sex, Ontario, Canada*

Characteristics	Mean (95% CI)			
	30 d	90 d	180 d	360 d
Total	$790 ($753–$826)	$2,043 ($1,949–$2,142)	$2,215 ($2,083–$2,346)	$2,553 ($2,348–$2,756)
Age, y				
<2†	$404 ($283–$526)	$1,094 ($756–$1,432)	$1,233 ($766–$1,701)	$1,512 ($786–$2,240)
2–4†	$59 ($20– $97)	$168 ($47–$290)	$273 ($−23 to $570)	$484 ($−161 to $1,132)
5–11†	$69 ($35–$104)	$185 ($89–$281)	$189 ($55–$323)	$196 ($−14 to $406)
12–17†	$45 ($21–$69)	$122 ($52–$193)	$143 ($18–$269)	$184 ($−50 to $420)
18–29	$89 ($72–$106)	$239 ($190–$288)	$259 ($179–$337)	$293 ($134–$449)
30–49	$348 ($311–$385)	$932 ($832–$1,015)	$1,030 ($888–$1,172)	$1,224 ($1,000–$1,449)
50–69	$1,366 ($1,27–$1,461)	$3,556 ($3,305–$3,656)	$3,789 ($3,454–$4,125)	$4,247 ($3,748–$4,745)
>70	$1,672 ($1,532–$1,813)	$3,958 ($3,633–$3,841)	$4,242 ($3,804–$4,680)	$13,841 ($12,193–$15,489)
Sex				
F	$613 ($572–$655)	$1,611 ($1,504–$1,753)	$1,807 ($1,651–$1,963)	$2,194 ($1,945–$2,446)
M	$971 ($911–$1,031)	$2,490 ($2,335–$2,603)	$2,637 ($2,426–$2,848)	$2,921 ($2,602–$3,241)

*Costs are rounded to the nearest dollar and presented in 2023 Canadian dollars. CI, confidence interval †Calculated as if no one died in these categories because of small cells or being unable to model a stable attributable cost estimate for the terminal phases.

## Discussion

We characterized the acute and long-term healthcare costs attributable to COVID-19 in Ontario, Canada, among persons who tested positive for SARS-CoV-2, compared with a group of persons who were unexposed to SARS-CoV-2. Exposed persons had higher costs than unexposed persons after the index date, and the largest difference occurred during the acute phase. Although the attributable costs were reduced in the postacute phase, mean costs remained greater among exposed persons compared with unexposed persons until the end of follow-up at 1-year post–index date.

The leading cost category in COVID-19–attributable cost across all phases was inpatient care, contributing 77% in the acute phase and 42% during the postacute phase. This result is consistent with descriptions of the natural history of SARS-CoV-2 acute infections and subsequent PCC. Acute infection often requires care to address acute symptoms, including potential respiratory distress, that would be best addressed in hospital, whereas PCC consists of several linked syndromic conditions are more likely to require complex care, rehabilitation, and outpatient visits (*34*).

We found the duration of acute illness to be 80 days. This duration is longer than durations reported in other studies (*14*) but lends empirical support to a growing global consensus that PCC is defined by symptoms that persist >12 weeks after infection onset (*35*–*37*).

The mean cumulative attributable cost for COVID-19 at 360 days was $2,553 per person testing positive for SARS-CoV-2, representing nearly half of the per capita healthcare spending in Ontario, which was $5,400 in 2020 (*38*). For the exposed cohort of 159,817 persons, this cost translates to $408 million. Meanwhile, the estimated cost for PCC ($18 attributable cost in the postacute phase per person/10 days) amounts to about $75 million for the exposed cohort over 1 year.

Our findings are comparable with growing evidence on COVID-19–associated cost. Previous publications have estimated healthcare costs associated with COVID-19 during the first wave of the pandemic in 2 provinces of Canada, British Columbia and Ontario (*39*), stratified by the level of initial care required, including community, long-term care, hospital, and ICU settings. In Ontario, the net costs (in 2020) during the first 120 days of diagnosis were $28,329 for persons who were hospitalized and $96,308 for those admitted to the ICU. Those estimates were comparable to those reported in the United States (*40**,**41*) and countries in Europe (*42*). In our study, after stratifying by the level of initial care received (Appendix Table 9), the net costs over a 360-day period were $30,147 for persons who were hospitalized and $105,677 for persons who were admitted to ICU. The higher costs in our study, because of the longer time length, suggest the ongoing effects of PCC, particularly among those with initially severe disease. Consistent with our estimated cost for PCC ($18 attributable cost per person for every 10 days in the postacute phase), a previous study in Ontario reported the mean attributable healthcare costs of $487 (95% CI $394–$593) in a matched cohort of adults starting >56 days after a positive SARS-CoV-2 PCR test over 1 year of follow-up (*15*). Two US studies examining costs up to 6 months by using data from private health insurance claims and Medicare beneficiaries showed that persons with COVID-19 had >1.4× higher direct medical costs after 31 days–6 months compared with matched controls (*43*), and, compared with historical controls, costs remained higher until the fifth month after diagnosis and were primarily driven by inpatient care (*44*), consistent with our findings. Further, we found that even after matching for comorbidities, frailty, and other factors, healthcare costs remained higher for persons living in lower-income neighborhoods. This finding aligns with a study conducted in Ontario, showing persons living in lower-income neighborhoods have a higher risk of death, a factor that likely contributes to increased end-of-life healthcare costs (*45*).

The first limitation of our study is that outcomes exclude costs not covered by Ontario’s public insurance system, such as medications for most persons <65 years of age, copayment costs covered by private insurance, or community-level services such as supportive housing. Second, although 96% of exposed persons were matched to unexposed persons, unmatched exposed persons were more likely to live in lower income neighborhoods, in urban areas, had higher ACG scores and were more likely to be frail compared with the matched exposed, making findings less generalizable to persons with these characteristics. Third, administrative data lack information to accurately define PCC, which is why we used costs incurred during the postacute phase as a proxy to estimate the economic burden attributable to PCC. However, as PCC diagnosis becomes more reliably captured within administrative datasets, future analyses could adopt a direct approach to estimate healthcare costs associated with PCC. Finally, our study reflects early pandemic experiences, when the likelihood of severe illness requiring hospitalization was higher, leading to increased healthcare costs. COVID-19 and associated healthcare costs may differ in contemporary times with the emergence of novel variants, vaccinations, treatments, and changes in clinical practice that were not assessed in this study.

Our study strengths included that because of Ontario’s public single-payer system, most of the community dwelling population was eligible for this study and the cohort was well matched. Of note, the absence of COVID-19–attributable costs at baseline suggested minimal residual confounding. Furthermore, matching to historic controls ensured that changes in healthcare delivery during the pandemic did not affect the unexposed group, which could bias attributable cost estimates for COVID-19. The use of several linked administrative databases enabled the characterization of COVID-19–attributable costs across a broad range of cost categories, providing a comprehensive view of COVID-19 healthcare costs. Finally, a phase-of-care approach enabled a comparison of acute versus postacute costs and the use of joinpoint regression enabled a data-driven approach to defining phase length.

Our findings offer insights into clinically relevant phase-specific costs, which can inform budget planning to ensure each healthcare sector is appropriately resourced. Inpatient care emerges as a major cost category, emphasizing the need for hospital resources. Of note, the costs associated with COVID-19 extend beyond the acute phase, indicating a growing effect of PCC on healthcare systems. This growth underscores the importance of allocating resources not only for immediate care but also for the long-term needs of persons with PCC, who will likely require ongoing access to healthcare services, including diagnostics, medications, and specialized treatments. Furthermore, our findings suggest that certain populations may experience disproportionate impacts, underscoring the need to address socioeconomic disparities through tailored healthcare policies. Policymakers can use those findings to prioritize investments and to assess the value of COVID-19 interventions to inform current policy decision-making and pandemic planning.

In conclusion, SARS-CoV-2 infection is associated with increased healthcare costs in the year following onset, with differential cost patterns in the acute and postacute phases, consistent with the evolving clinical understanding of PCC. Our findings have major implications for stakeholders responding to PCC at the health system level. Determining phase-specific healthcare costs for SARS-CoV-2 infection can inform future health sector budget and pandemic planning.

Appendix Additional information for population-based matched cohort study of COVID-19 healthcare costs, Ontario, Canada.

## References

[1] Canadian Institute for Health Information. Impact of COVID-19 on Canada’s health care systems. 2023 [cited 2024 Jun 17]. https://www.cihi.ca/en/covid-19-resources/impact-of-covid-19-on-canadas-health-care-systems

[2] Quinn KL, Katz GM, Bobos P, Sander B, McNaughton CD, Cheung AM, et al. Understanding the post COVID-19 condition (long COVID) in adults and the expected burden for Ontario. Science Briefs of the Ontario COVID-19 Science Advisory Table. 2022;3(65).

[3] Katz GM, Bach K, Bobos P, Cheung A, Décary S, Goulding S, et al. Understanding how post–COVID-19 condition affects adults and health care systems. JAMA Health Forum. 2023;4:e231933. 10.1001/jamahealthforum.2023.1933 37418268

[4] World Health Organization. Post COVID-19 condition, long COVID. 2022 [cited 2024 Jun 17]. https://www.who.int/europe/news-room/fact-sheets/item/post-covid-19-condition

[5] Blanchflower DG, Bryson A. Long COVID in the United States. PLoS One. 2023;18:e0292672. 10.1371/journal.pone.0292672 37917610 PMC10621843

[6] Government of Canada. COVID-19 variants: wastewater monitoring dashboard [cited 2024 Dec 12]. https://health-infobase.canada.ca/wastewater

[7] McNaughton CD, Austin PC, Sivaswamy A, Fang J, Abdel-Qadir H, Daneman N, et al. Post-acute health care burden after SARS-CoV-2 infection: a retrospective cohort study. CMAJ. 2022;194:E1368–76. 10.1503/cmaj.220728 36252983 PMC9616149

[8] Tartof SY, Malden DE, Liu IA, Sy LS, Lewin BJ, Williams JTB, et al. Health care utilization in the 6 months following SARS-CoV-2 infection. JAMA Netw Open. 2022;5:e2225657. 10.1001/jamanetworkopen.2022.25657 35960522 PMC9375168

[9] Whittaker HR, Gulea C, Koteci A, Kallis C, Morgan AD, Iwundu C, et al. GP consultation rates for sequelae after acute covid-19 in patients managed in the community or hospital in the UK: population based study. BMJ. 2021;375:e065834. 10.1136/bmj-2021-065834 34965929 PMC8715128

[10] Patterson B, Ruppenkamp J, Richards F, Debnath R, ElKhoury AC, DeMartino JK, et al. Cost of long COVID following severe disease a US healthcare database analysis. Value Health. 2022;25:S375. 10.1016/j.jval.2022.04.460

[11] The COVID-19 Immunity Task Force. Seroprevalence in Canada [cited 2024 Jun 17]. https://www.covid19immunitytaskforce.ca/seroprevalence-in-canada

[12] Shrestha SS, Kompaniyets L, Grosse SD, Harris AM, Baggs J, Sircar K, et al. Estimation of coronavirus disease 2019 hospitalization costs from a large electronic administrative discharge database, March 2020–July 2021. Open Forum Infect Dis. 2021;8:ofab561. 10.1093/ofid/ofab561 34938822 PMC8686820

[13] Yang J, Andersen KM, Rai KK, Tritton T, Mugwagwa T, Reimbaeva M, et al. Healthcare resource utilisation and costs of hospitalisation and primary care among adults with COVID-19 in England: a population-based cohort study. BMJ Open. 2023;13:e075495. 10.1136/bmjopen-2023-075495 38154885 PMC10759085

[14] Wolff Sagy Y, Feldhamer I, Brammli-Greenberg S, Lavie G. Estimating the economic burden of long-Covid: the additive cost of healthcare utilisation among COVID-19 recoverees in Israel. BMJ Glob Health. 2023;8:e012588. 10.1136/bmjgh-2023-012588 37463787 PMC10357303

[15] McNaughton CD, Austin PC, Li Z, Sivaswamy A, Fang J, Abdel-Qadir H, et al. Higher post-acute health care costs following SARS-CoV-2 infection among adults in Ontario, Canada. J Multidiscip Healthc. 2024;17:5749–61. 10.2147/JMDH.S465154 39659735 PMC11628314

[16] Benchimol EI, Smeeth L, Guttmann A, Harron K, Moher D, Petersen I, et al.; RECORD Working Committee. The REporting of studies Conducted using Observational Routinely-collected health Data (RECORD) statement. PLoS Med. 2015;12:e1001885. 10.1371/journal.pmed.1001885 26440803 PMC4595218

[17] Government of Canada. COVID-19 vaccination: doses administered [cited 2024 Jun 17]. https://health-infobase.canada.ca/covid-19/vaccine-administration

[18] Dave N, Sjöholm D, Hedberg P, Ternhag A, Granath F, Verberk JDM, et al. Nosocomial SARS-CoV-2 infections and mortality during unique COVID-19 epidemic waves. JAMA Netw Open. 2023;6:e2341936. 10.1001/jamanetworkopen.2023.41936 37948082 PMC10638644

[19] Mitchell R, Choi KB, Pelude L, Rudnick W, Thampi N, Taylor G; CNISP COVID-19 Working Group. Patients in hospital with laboratory-confirmed COVID-19 in a network of Canadian acute care hospitals, Mar. 1 to Aug. 31, 2020: a descriptive analysis. CMAJ Open. 2021;9:E149–56. 10.9778/cmajo.20200246 33653770 PMC8034374

[20] Ontario Agency for Health Protection and Promotion; Provincial Infectious Diseases Advisory Committee on Infection Prevention and Control. Best practices for the prevention of acute respiratory infection transmission in all health care settings, 2024 [cited 2025 Mar 3]. https://www.publichealthontario.ca/-/media/Documents/A/24/acute-respiratory-infection-transmission.pdf

[21] Zeitouny S, Cheung DC, Bremner KE, Pataky RE, Pequeno P, Matelski J, et al. The impact of the early COVID-19 pandemic on healthcare system resource use and costs in two provinces in Canada: An interrupted time series analysis. PLoS One. 2023;18:e0290646. 10.1371/journal.pone.0290646 37682823 PMC10490868

[22] Canadian Institute for Health Information. COVID-19 hospitalization and emergency department statistics, 2020–2021, August 2022 [cited 2025 Mar 3]. https://www.cihi.ca/en/covid-19-hospitalization-and-emergency-department-statistics

[23] Glazier RH, Green ME, Wu FC, Frymire E, Kopp A, Kiran T. Shifts in office and virtual primary care during the early COVID-19 pandemic in Ontario, Canada. CMAJ. 2021;193:E200–10. 10.1503/cmaj.202303 33558406 PMC7954541

[24] Public Health Ontario. The story of COVID-19 testing in Ontario [cited 2024 Jun 25]. https://www.publichealthontario.ca/en/About/News/2020/Story-Covid-19-Testing-Ontario

[25] Austin PC. An introduction to propensity score methods for reducing the effects of confounding in observational studies. Multivariate Behav Res. 2011;46:399–424. 10.1080/00273171.2011.568786 21818162 PMC3144483

[26] Austin PC. Optimal caliper widths for propensity-score matching when estimating differences in means and differences in proportions in observational studies. Pharm Stat. 2011;10:150–61. 10.1002/pst.433 20925139 PMC3120982

[27] The Johns Hopkins Bloomberg School of Public Health. The Johns Hopkins ACG® System. Excerpt from version 13.0 technical reference guide, June 2022 [cited 2024 Jul 24]. https://www.healthpartners.com/content/dam/brand-identity/pdfs/care/acg-technical-guide.pdf

[28] Johns Hopkins Medicine. The ACG system is an essential tool in helping providers manage multiple chronic conditions [cited 2024 Jul 24]. https://www.hopkinsacg.org/the-acg-system-is-an-essential-tool-in-helping-providers-manage-multiple-chronic-conditions

[29] Matheson FI, Moloney G, van Ingen T; Public Health Ontario. Ontario marginalization index: user guide, 2023 [cited 2025 Mar 3]. https://www.publichealthontario.ca/-/media/documents/o/2017/on-marg-userguide.pdf 10.17269/s41997-021-00552-1 PMC8975983 34432255

[30] Tanuseputro P, Wodchis WP, Fowler R, Walker P, Bai YQ, Bronskill SE, et al. The health care cost of dying: a population-based retrospective cohort study of the last year of life in Ontario, Canada. PLoS One. 2015;10:e0121759. 10.1371/journal.pone.0121759 25811195 PMC4374686

[31] Wodchis WP, Bushmeneva K, Nikitovic M, Mckillop I. Guidelines on person-level costing using administrative databases in Ontario, 2013 [cited 2024 Nov 20]. http://www.hsprn.ca/uploads/files/Guidelines_on_PersonLevel_Costing_May_2013.pdf

[32] Yabroff KR, Lamont EB, Mariotto A, Warren JL, Topor M, Meekins A, et al. Cost of care for elderly cancer patients in the United States. J Natl Cancer Inst. 2008;100:630–41. 10.1093/jnci/djn103 18445825

[33] Kim HJ, Fay MP, Feuer EJ, Midthune DN. Permutation tests for joinpoint regression with applications to cancer rates. Stat Med. 2000;19:335–51. 10.1002/(SICI)1097-0258(20000215)19:3<335::AID-SIM336>3.0.CO;2-Z 10649300

[34] Parotto M, Gyöngyösi M, Howe K, Myatra SN, Ranzani O, Shankar-Hari M, et al. Post-acute sequelae of COVID-19: understanding and addressing the burden of multisystem manifestations. Lancet Respir Med. 2023;11:739–54. 10.1016/S2213-2600(23)00239-4 37475125

[35] World Health Organization. A clinical case definition of post COVID-19 condition by a Delphi consensus, 6 October 2021 [cited 2024 Jun 14]. https://www.who.int/publications/i/item/WHO-2019-nCoV-Post_COVID-19_condition-Clinical_case_definition-2021.1

[36] Soriano JB, Murthy S, Marshall JC, Relan P, Diaz JV; WHO Clinical Case Definition Working Group on Post-COVID-19 Condition. A clinical case definition of post-COVID-19 condition by a Delphi consensus. Lancet Infect Dis. 2022;22:e102–7. 10.1016/S1473-3099(21)00703-9 34951953 PMC8691845

[37] Centers for Disease Control and Prevention. Clinical overview of long COVID [cited 2024 Jun 24]. https://www.cdc.gov/coronavirus/2019-ncov/hcp/clinical-care/post-covid-conditions.html

[38] Statistics Canada. Expenses of government classified by function, 2020 [cited 2025 Mar 3]. https://www150.statcan.gc.ca/n1/en/daily-quotidien/211126/dq211126a-eng.pdf?st=EL7ygEmS

[39] Tsui TCO, Zeitouny S, Bremner KE, Cheung DC, Mulder C, Croxford R, et al. Initial health care costs for COVID-19 in British Columbia and Ontario, Canada: an interprovincial population-based cohort study. CMAJ Open. 2022;10:E818–30. 10.9778/cmajo.20210328 36126976 PMC9497846

[40] Tsai Y, Vogt TM, Zhou F. Patient characteristics and costs associated with covid-19-related medical care among medicare fee-for-service beneficiaries. Ann Intern Med. 2021;174:1101–9. 10.7326/M21-1102 34058109 PMC8252832

[41] Kapinos KA, Peters RM Jr, Murphy RE, Hohmann SF, Podichetty A, Greenberg RS. Inpatient costs of treating patients with COVID-19. JAMA Netw Open. 2024;7:e2350145. 10.1001/jamanetworkopen.2023.50145 38170519 PMC10765267

[42] Czernichow S, Bain SC, Capehorn M, Bøgelund M, Madsen ME, Yssing C, et al. Costs of the COVID-19 pandemic associated with obesity in Europe: A health-care cost model. Clin Obes. 2021;11:e12442. 10.1111/cob.12442 33554456 PMC7988570

[43] Pike J, Kompaniyets L, Lindley MC, Saydah S, Miller G. Direct medical costs associated with post–COVID-19 conditions among privately insured children and adults. Prev Chronic Dis. 2023;20:E06. 10.5888/pcd20.220292 36757854 PMC9923935

[44] DeMartino JK, Swallow E, Goldschmidt D, Yang K, Viola M, Radtke T, et al. Direct health care costs associated with COVID-19 in the United States. J Manag Care Spec Pharm. 2022;28:936–47. 10.18553/jmcp.2022.22050 35722829 PMC12101566

[45] Wang L, Calzavara A, Baral S, Smylie J, Chan AK, Sander B, et al. Differential patterns by area-level social determinants of health in coronavirus disease 2019 (COVID-19)-related mortality and non–COVID-19 mortality: a population-based study of 11.8 million people in Ontario, Canada. Clin Infect Dis. 2023;76:1110–20. 10.1093/cid/ciac850 36303410 PMC9620355

