# Supplementary material for: Population-Based Matched Cohort Study of COVID-19 Healthcare Costs, Ontario, Canada
Source: Emerg Infect Dis. 2025 Apr;31(4):710–9. doi: 10.3201/eid3104.241463 (PMC11950279; doi:10.3201/eid3104.241463)
Supplement: Appendix — Additional information for population-based matched cohort study of COVID-19 healthcare costs, Ontario, Canada. [file 24-1463-Techapp-s1.pdf]

Article DOI: https://doi.org/10.3201/eid3104.241463

EID cannot ensure accessibility for supplementary materials supplied by authors. Readers who have difficulty accessing supplementary content should contact the authors for assistance.

Population-Based Matched Cohort Study of COVID-19 Healthcare Costs, Ontario, Canada

Appendix

Appendix Table 1. ICES Datasets used in this study

List of datasets	Full name	Description
C19INTGR	COVID19 Integrated Testing Data	Used to determine those with a positive C19 infection
CCM	Case and Contact Management System	Used to determine those with a positive C19 infection
CCRS	Continuing Care Reporting System	Used to determine those living in Long-Term care facilities and part of the healthcare cost calculation
CENSUS	Ontario Census Area Profiles	Used to create the Essential worker quintile
DAD	Discharge Abstract Database DAD	Part of the health care cost calculations and hospitalizations
HCD	Home Care Database	Part of the health care cost calculations
CIC/IRCC	IRCC Permanent Residents database	Used to determine immigration status
NACRS	National Ambulatory Care Reporting System	Part of the health care cost calculations
ODB	Ontario Drug Benefit Claims	Part of the health care cost calculations
OHIP	Ontario Health Insurance Plan Claims Database	Part of the health care cost calculations
OLISC19	OLIS COVID-19 Laboratory Data	Used to determine those with a positive C19 infection
OMHRS	Ontario Mental Health Reporting System	Part of the health care cost calculations
ON-MARG	Ontario Marginalization Index	Used to create the Age and labor force quintiles, Material resource quintiles, Racialized and newcomer populations quintile, and Households and dwellings quintile
PCCF	Postal Code Conversion File	Used to determine postal code for Ontario residency
RPDB	Registered Persons Database	Demographics information on birth date, sex, ohip eligibility, date of last contact with the healthcare system, and death date
SDS	Same Day Surgery Database (Annual)	Part of the health care cost calculations
ACG	ACG macro	Used in combination with OHIP, DAD, and NACRS to calculate ACGs, RUB, and frailty
NRS	National Rehabilitation Reporting System	Part of the health care cost calculations
NDFP	New Drug Funding Program	Part of the health care cost calculations
ADP	Assistive Devices Program	Part of the health care cost calculations
INST	Facilities	Used to determine those living in Long-Term care facilities

Appendix Table 2. Demographics of individuals exposed to COVID-19 and unexposed individuals in the terminal phase

	Matched			Unmatched
**Characteristics**	**Exposed N = 3,114**	**Unexposed N = 3,114**	**SMD**	**Exposed N = 243**
Age at index, years, mean (sd)	76.9 ± 14.6	75.4 ± 16.1	0.10	81.7 ± 16.6
Female sex, n (%)	1,399 (44.9)	1,399 (44.9)	0.00	109 (44.9)
Rural, n (%)	116 (3.7)	104 (3.3)	0.02	< = 5 (...)
Immigrant, n (%)	720 (23.1)	720 (23.1)	0.00	83 (34.2)
Frail, n (%)	1,056 (33.9)	1,136 (36.5)	0.05	136 (56.0)
ACG, median (IQR)	9.0 (6.0–12.0)	9.0 (6.0–12.0)	0.09	9.0 (6.0–12.0)
**Neighborhood income quintile, n (%)**				
1st quintile (lowest)	889 (28.5)	889 (28.5)	0.00	68 (28.0)
2nd quintile	738 (23.7)	738 (23.7)	0.00	39 (16.0)
3rd quintile	631 (20.3)	631 (20.3)	0.00	78 (32.1)
4th quintile	435 (14.0)	435 (14.0)	0.00	22 (9.1)
5th quintile (highest)	421 (13.5)	421 (13.5)	0.00	36 (14.8)

Characteristics	Matched Exposed N = 3,114	Matched Unexposed N = 3,114	SMD	Unmatched Exposed N = 243
**Age and labor force quintile, n (%)**				
1st quintile (lowest)	619 (19.9)	602 (19.3)	0.01	58 (23.9)
2nd quintile	577 (18.5)	584 (18.8)	0.01	49 (20.2)
3rd quintile	542 (17.4)	550 (17.7)	0.01	22 (9.1)
4th quintile	533 (17.1)	559 (18.0)	0.02	73 (30.0)
5th quintile (highest)	823 (26.4)	803 (25.8)	0.01	35 (14.4)
**Material resource quintile, n (%)**				
1st quintile (lowest)	529 (17.0)	550 (17.7)	0.02	44 (18.1)
2nd quintile	513 (16.5)	503 (16.2)	0.01	44 (18.1)
3rd quintile	565 (18.1)	574 (18.4)	0.01	62 (25.5)
4th quintile	654 (21.0)	633 (20.3)	0.02	38 (15.6)
5th quintile	833 (26.8)	838 (26.9)	0.00	49 (20.2)
**Racialized and newcomer populations quintile, n (%)**				
1st quintile (lowest)	267 (8.6)	242 (7.8)	0.03	19 (7.8)
2nd quintile	371 (11.9)	339 (10.9)	0.03	40 (16.5)
3rd quintile	488 (15.7)	495 (15.9)	0.01	31 (12.8)
4th quintile	680 (21.8)	693 (22.3)	0.01	47 (19.3)
5th quintile	1,288 (41.4)	1,329 (42.7)	0.03	100 (41.2)
**Households and dwellings quintile, n (%)**				
1st quintile (lowest)	541 (17.4)	549 (17.6)	0.01	51 (21.0)
2nd quintile	442 (14.2)	442 (14.2)	0.00	22 (9.1)
3rd quintile	491 (15.8)	478 (15.4)	0.01	33 (13.6)
4th quintile	557 (17.9)	528 (17.0)	0.02	46 (18.9)
5th quintile	1,063 (34.1)	1,101 (35.4)	0.03	85 (35.0)
**Essential worker quintile, n (%)**				
1st quintile (lowest)	608 (19.5)	616 (19.8)	0.01	38 (15.6)
2nd quintile	601 (19.3)	599 (19.2)	0.00	67 (27.6)
3rd quintile	612 (19.7)	594 (19.1)	0.01	30 (12.3)
4th quintile	654 (21.0)	654 (21.0)	0.00	55 (22.6)
5th quintile	631 (20.3)	636 (20.4)	0.00	49 (20.2)
**Resource utilization band, n (%)**				
Non-users	35 (1.1)	35 (1.1)	0.00	12 (4.9) [a]
Healthy Users	10 (0.3)	10 (0.3)	0.00	
Low resource utilization	79 (2.5)	79 (2.5)	0.00	12 (4.9)
Moderate resource utilization	832 (26.7)	832 (26.7)	0.00	54 (22.2)
High resource utilization	658 (21.1)	658 (21.1)	0.00	52 (21.4)
Very high resource utilization	1,500 (48.2)	1,500 (48.2)	0.00	113 (46.5)

ACG: adjusted clinical groups; IQR; interquartile range; N: number of observations, sd: standard deviation, SMD: standardized difference in means
[a] two categories were combined because of small number

Appendix Table 3. Total and COVID-19–attributable healthcare costs standardized to 10 days by phase of care, Ontario Canada

Cost categories	Exposed n = 159,817	Unexposed n = 159,817	Net cost n = 159,817
Prediagnosis phase (30 d before index date)			
Total costs, mean (95%CI)	$ 106.70 (103.20, 110.20)	$ 106.00 (102.60, 109.40)	$ 1.10 (−3.50, 5.70)
Costs by resource category, mean (95% CI)			
Inpatient care	$ 24.30 (22.20, 26.50)	$ 25.80 (23.40, 28.20)	$ −1.40 (−4.50, 1.70)
Outpatient services	$ 16.60 (15.80, 17.50)	$ 15.40 (14.70, 16.00)	$ 1.30 (0.20, 2.40)
ED visits	$ 5.60 (5.40, 5.80)	$ 3.70 (3.60, 3.80)	$ 1.90 (1.70, 2.20)
Medications	$ 11.80 (11.20, 12.30)	$ 12.20 (11.50, 13.00)	$ −0.50 (−1.40, 0.50)
Physician services	$ 6.90 (6.80, 7.00)	$ 7.50 (7.40, 7.60)	$ −0.50 (−0.70, −0.40)
Rehabilitation	$ 3.10 (2.40, 3.90)	$ 2.00 (1.40, 2.50)	$ 1.20 (0.20, 2.10)
Complex care	$ 4.30 (3.40, 5.20)	$ 3.20 (2.50, 3.90)	$ 1.10 (0.00, 2.30)
Homecare	$ 8.90 (8.30, 9.50)	$ 8.20 (7.70, 8.60)	$ 0.80 (0.10, 1.60)
Long term care	$ 0.00 (.,.)	$ 0.00 (.,.)	$ 0.00 (.,.)
Other	$ 24.30 (22.20, 26.50)	$ 25.80 (23.40, 28.20)	$ −1.40 (−4.50, 1.70)
Acute phase (80 d including index)			
Total costs, mean (95%CI)	$ 334.20 (325.00, 343.30)	$ 94.20 (91.80, 96.70)	$ 240.10 (230.80, 249.40)
Costs by resource category, mean (95% CI)			
Inpatient care	$ 205.50 (197.80, 213.20)	$ 21.10 (19.50, 22.60)	$ 184.50 (176.60, 192.30)
Outpatient services	$ 18.60 (17.90, 19.30)	$ 14.60 (13.90, 15.20)	$ 4.00 (3.10, 5.00)
ED visits	$ 18.80 (18.50, 19.10)	$ 3.50 (3.40, 3.60)	$ 15.30 (15.00, 15.60)
Medications	$ 11.10 (10.80, 11.50)	$ 11.70 (11.10, 12.20)	$ −0.50 (−1.10, 0.10)
Physician services	$ 8.10 (8.00, 8.20)	$ 5.50 (5.40, 5.60)	$ 2.60 (2.50, 2.70)
Rehabilitation	$ 7.90 (7.10, 8.70)	$ 1.50 (1.20, 1.80)	$ 6.40 (5.50, 7.20)
Complex care	$ 6.80 (5.90, 7.70)	$ 3.30 (2.60, 4.00)	$ 3.50 (2.40, 4.60)

Cost categories	Exposed n = 159,817	Unexposed n = 159,817	Net cost n = 159,817
Homecare	$ 9.80 (9.30, 10.30)	$ 7.40 (7.00, 7.80)	$ 2.50 (1.90, 3.10)
Long term care	$ 0.50 (0.40, 0.60)	$ 0.60 (0.50, 0.80)	$ −0.20 (−0.40, 0.10)
Other	$ 1.70 (1.60, 1.70)	$ 2.60 (2.60, 2.70)	$ −1.00 (−1.00, −0.90)
Post-acute phase (81 to 360 d post index or to the start of the terminal phase, whichever occurred first)			
Total costs, mean (95%CI)	$ 112.10 (109.10, 115.20)	$ 95.10 (92.90, 97.20)	$ 17.50 (13.90, 21.00)
Costs by resource category, mean (95% CI)			
Inpatient care	$ 29.10 (27.00, 31.10)	$ 21.80 (20.50, 23.10)	$ 7.30 (4.90, 9.60)
Outpatient services	$ 18.00 (17.30, 18.70)	$ 14.90 (14.40, 15.50)	$ 3.10 (2.20, 4.00)
ED visits	$ 4.70 (4.60, 4.80)	$ 3.60 (3.50, 3.70)	$ 1.10 (1.00, 1.30)
Medications	$ 12.20 (11.70, 12.60)	$ 11.80 (11.40, 12.30)	$ 0.30 (−0.30, 1.00)
Physician services	$ 4.50 (4.40, 4.60)	$ 4.70 (4.60, 4.70)	$ −0.20 (−0.20,-0.10)
Rehabilitation	$ 1.70 (1.40, 1.90)	$ 1.30 (1.10, 1.60)	$ 0.30 (0.00, 0.60)
Complex care	$ 4.70 (4.00, 5.40)	$ 2.80 (2.20, 3.30)	$ 1.90 (1.00, 2.80)
Homecare	$ 8.40 (7.90, 8.90)	$ 7.00 (6.60, 7.40)	$ 1.40 (0.80, 2.00)
Long term care	$ 4.70 (4.10, 5.20)	$ 3.10 (2.70, 3.50)	$ 1.50 (0.90, 2.20)
Other	$ 2.00 (2.00, 2.00)	$ 2.50 (2.50, 2.50)	$ −0.50 (−0.50, −0.50)
Terminal phase, early deaths (deaths within 60 d of index)			
	Exposed n = 3,114	Unexposed n = 3,114	Net cost n = 3,114
Total costs, mean (95%CI)	$ 8,723.60 (8,328.30, 9,118.90)	$ 4,796.10 (4,550.90, 5,041.30)	$ 3,927.50 (3,471.20, 4,383.80)
Costs by resource category, mean (95% CI)			
Inpatient care	$ 7,051.80 (6,680.80, 7,422.80)	$ 2,820.10 (2,607.00, 3,033.20)	$ 4,231.70 (3,813.30, 4,650.10)
Outpatient services	$ 253.80 (229.10, 278.50)	$ 273.40 (247.20, 299.50)	$ −19.50 (−54.80, 15.70)
ED visits	$ 184.00 (178.60, 189.40)	$ 123.40 (117.70, 129.10)	$ 60.60 (52.90, 68.30)
Medications	$ 102.20 (88.30, 116.20)	$ 119.30 (103.10, 135.40)	$ −17.00 (−38.10, 4.10)
Physician services	$ 44.70 (42.20, 47.10)	$ 52.60 (48.90, 56.30)	$ −7.90 (−12.30, −3.60)
Rehabilitation	$ 74.90 (46.80, 102.90)	$ 54.40 (33.20, 75.50)	$ 20.50 (−14.80, 55.80)
Complex care	$ 102.40 (69.00, 135.70)	$ 252.40 (202.20, 302.60)	$ −150.00 (−210.30, −89.80)
Homecare	$ 209.40 (186.50, 232.30)	$ 365.40 (340.20, 390.50)	$-155.90 (−189.10, −122.80)
Long term care	$ 1.00 (−0.50, 2.60)	$ 334.50 (288.90, 380.10)	$ −333.50 (−379.00, −288.00)
Other	$ 2.90 (2.60, 3.10)	$ 4.70 (4.30, 5.10)	$ −1.80 (−2.30, −1.30)
Terminal phase, late deaths (deaths after 60 d of index)			
Total costs, mean (95%CI)	$ 6,708.70 (6,193.60, 7,223.70)	$ 4,927.70 (4602.60, 5252.80)	$ 1,780.90 (1,181.90, 2379.90)
Costs by resource category, mean (95% CI)			
Inpatient care	$ 4,386.30 (3,909.30, 4,863.30)	$ 2,828.70 (2547.30, 3110.00)	$ 1,557.60 (1,010.60, 2,104.70)
Outpatient services	$ 398.90 (351.60, 446.20)	$ 302.00 (265.70, 338.20)	$ 96.90 (38.70, 155.20)
ED visits	$ 144.90 (135.40, 154.50)	$ 123.90 (116.40, 131.40)	$ 21.00 (8.80, 33.30)
Medications	$ 138.90 (118.60, 159.20)	$ 119.30 (104.00, 134.60)	$ 19.60 (−60.00, 45.20)
Physician services	$ 63.80 (58.50, 69.10)	62.30 (54.70, 69.80)	$ 1.50 (−7.20, 10.30)
Rehabilitation	$ 45.30 (22.30, 68.30)	$ 51.80 (16.00, 87.50)	$ −6.50 (−43.90, 30.90)
Complex care	$ 332.50 (254.30, 410.80)	$ 305.00 (233.40, 376.70)	$ 27.50 (−76.80, 131.80)
Homecare	$ 408.30 (367.50, 449.10)	$ 375.80 (337.80, 413.70)	$ 32.50 (−22.30, 87.40)
Long term care	$ 295.70 (241.00, 350.30)	$ 368.80 (309.10, 428.50)	$ −73.10 (−151.60, 5.40)
Other	$ 4.60 (4.00, 5.30)	$ 5.20 (4.50, 5.80)	$ −0.50 (−1.50, 0.40)

*CI, confidence interval; ED, emergency department.
Costs are in Canadian dollars

Appendix Table 4. Total and COVID-19-attributable healthcare costs (2023 CAD) standardized to 10 d stratified by age

Age categories	N	Exposed mean (95% CI)	N	Unexposed mean (95% CI)	Net cost mean (95% CI)
**Prediagnosis phase (30 d before index date)**					
0 to <2 y old*	1,138	$ 100.90 (70.10, 131.70)	1,139	$ 163.30 (95.40, 231.20)	$ −62.40 (−137.10, 12.20)
2–4 y old*	2,152	$ 27.00 (16.80, 37.20)	2,152	$ 39.90 (28.50, 51.30)	$ −13.00 (−27.90, 2.00)
5–11 y old*	6,675	$ 23.70 (17.90, 29.50)	6,675	$ 23.30 (20.30, 26.20)	$ 0.40 (−5.90, 6.70)
12–17 y old*	8,519	$ 32.00 (26.50, 37.50)	8,519	$ 34.00 (28.30, 39.60)	$ −2.00 (−9.40, 5.50)
18–29 y old	35,515	$ 40.20 (36.80, 43.70)	35,517	$ 44.90 (41.40, 48.30)	$ −4.60 (−9.20, 0.00)
30–49 y old	51,773	$ 64.70 (60.90, 68.50)	51,809	$ 68.70 (64.90, 72.50)	$ −4.00 (−9.30, 1.20)
50–69 y old	41,601	$ 132.20 (124.4, 140.10)	41,903	$ 133.30 (125.90, 140.80)	$ −1.00 (−11.50, 9.40)
70+ years old	10,820	$ 553.90 (520.80, 586.90)	12,026	$ 455.20 (424.70, 485.60)	$ 100.40 (57.90, 143.00)
**Acute phase (80 d including index)**					
0 to <2 y old*	1,138	$ 189.20 (150.40, 227.90)	1,139	$ 54.40 (41.70, 67.10)	$ 134.80 (94.40, 175.30)
2–4 y old*	2,152	$ 49.50 (37.40, 61.50)	2,152	$ 29.90 (24.10, 35.80)	$ 19.50 (6.80, 32.30)
5–11 y old*	6,675	$ 45.00 (33.80, 56.20)	6,675	$ 21.90 (19.40, 24.50)	$ 23.10 (11.60, 34.60)
12–17 y old*	8,519	$ 47.70 (40.8, 54.50)	8,519	$ 32.60 (28.30, 36.90)	$ 15.00 (7.00, 23.10)
18–29 y old	35,512	$ 70.60 (65.50, 75.60)	35,517	$ 41.00 (38.20, 43.70)	$ 29.60 (23.90, 35.30)
30–49 y old	51,762	$ 172.70 (162.50, 183.00)	51,799	$ 59.70 (56.90, 62.50)	$ 113.00 (102.40, 123.60)
50–69 y old	41,484	$ 534.90 (509.90, 559.90)	41,887	$ 120.50 (115.00, 126.00)	$ 414.40 (389.00, 439.80)
70+ years old	10,563	$ 1,710.60 (1,638.50, 1,782.70)	11,969	$ 408.90 (388.10, 429.70)	$ 1,302.30 (1,228.50, 1,376.10)
**Post-acute phase (81 to 360 d post index or to the start of the terminal phase, whichever occurred first)**					
0 to <2 y old*	1,138	$ 49.20 (35.10, 63.20)	1,139	$ 33.70 (29.90, 37.50)	$ 15.50 (1.10, 29.90)
2–4 y old*	2,152	$ 39.30 (19.60, 59.10)	2,152	$ 27.60 (23.00, 32.20)	$ 11.70 (−7.70, 31.20)
5–11 y old*	6,675	$ 21.40 (17.80, 24.90)	6,675	$ 21.00 (18.80, 23.20)	$ 0.40 (−3.80, 4.60)
12–17 y old*	8,519	$ 33.40 (28.10, 38.60)	8,519	$ 31.00 (27.80, 34.30)	$ 2.30 (−3.80, 8.40)
18–29 y old	35,507	$ 43.40 (40.90, 45.90)	35,514	$ 41.20 (38.90, 43.50)	$ 2.20 (−1.20, 5.50)
30–49 y old	51,751	$ 70.20 (66.60, 73.80)	51,791	$ 59.70 (57.40, 61.90)	$ 10.50 (6.30, 14.70)
50–69 y old	41,421	$ 146.40 (139.40, 153.40)	41,841	$ 122.70 (118.00, 127.30)	$ 23.70 (15.60, 31.80)
70+ years old	10,373	$ 565.10 (536.90, 593.30)	11,806	$ 421.00 (401.60, 440.30)	$ 145.70 (112.70, 178.70)
**Terminal phase, early deaths (deaths within 60 d of index)** [a]					
18–29 y old	8	$ 7,600.50 (130.30, 15,070.80)	29	$ 4,299.10 (1,223.30, 7,374.90)	$ −1,387.10 (−8,157.30, 5,383.00)
30–49 y old	52	$ 10,7350 (7,478.10, 13,991.80)	106	$ 4,217.90 (2,880.80, 5,555.00)	$ 6,506.60 (3,089.80, 9,923.40)
50–69 y old	426	$ 13,465.30 (12,415.00, 14,515.50)	430	$ 5,244.40 (4,721.50, 5,767.40)	$ 8,219.80 (7,045.80, 9,393.70)
70+ years old	1,432	$ 7,251.60 (6,870.40, 7,632.70)	1,348	$ 4,689.00 (4,411.50, 4,966.40)	$ 2,541.40 (2,089.20, 2,993.60)
**Terminal phase, late deaths (deaths after 60 d of index)** [a]					
18–29 y old	21	$ 4,430.30 (1,272.10, 7,588.60)	22	$ 6,667.60 (1,846.70, 11,488.40)	$ −1,720.40 (−6,876.40, 3,435.60)
30–49 y old	56	$ 7,765.40 (4,569.20, 10,961.70)	60	$ 4,758.60 (3,430.90, 6,086.30)	$ 2,991.30 (−433.60, 6,416.30)
50–69 y old	278	$ 8,612.10 (7,302.50, 9,921.80)	257	$ 5,710.30 (4,789.40, 6631.10)	$ 2,915.00 (1,355.10, 4,474.90)
70+ years old	838	$ 6,070.60 (5,526.40, 6,614.80)	851	$ 4,611.60 (4,288.10, 4,935.10)	$ 1,451.40 (830.20, 2,072.60)

CI: confidence interval

[a] unable to model a stable attributable cost estimate for age <18 y due to small cells for terminal phases

Appendix Table 5. Total and COVID-19-attributable healthcare costs (2023 CAD) standardized to 10 d stratified by sex

Sex	N	Exposed mean (95% CI)	N	Unexposed mean (95% CI)	Net cost mean (95% CI)
**Prediagnosis phase (30 d before index date)**					
Female	80,315	$ 116.70 (111.70, 121.80)	80,946	$ 115.00 (110.00, 119.90)	$ 2.20 (−4.40, 8.80)
Male	77,878	$ 96.30 (91.50, 101.10)	78,794	$ 96.80 (92.00, 101.60)	$ −0.10 (−6.50, 6.40)
**Acute phase (80 d including index)**					
Female	80,182	$ 292.90 (282.40, 303.50)	80,899	$ 100.10 (96.70, 103.40)	$ 192.90 (182.20, 203.70)
Male	77,623	$ 376.80 (361.70, 391.80)	78,758	$ 88.20 (84.60, 91.80)	$ 288.7 (273.50, 304.0)
**Post-acute phase (81 to 360 d post index or to the start of the terminal phase, whichever occurred first)**					
Female	80,058	$ 119.90 (115.80, 123.90)	80,792	$ 99.30 (96.50, 102.10)	$ 20.80 (16.20, 25.50)
Male	77,478	$ 104.10 (99.70, 108.60)	78,645	$ 90.70 (87.40, 94.00)	$ 13.90 (8.60, 19.20)
**Terminal phase, early deaths (deaths within 60 d of index)**					
Female	773	7,058.90 (6,514.10, 7,603.60)	773	4,506.00 (4,154.30, 4,857.60)	$ 2,552.90 (1,918.00, 3,187.80)
Male	1,146	9,846.50 (9,304.90, 10,388.20)	1,146	4,991.80 (4,656.70, 5,326.90)	$ 4,854.70 (4,227.60, 5,481.80)
**Terminal phase, late deaths (deaths after 60 d of index)**					
Female	626	$ 5,774.10 (5,194.90, 6,353.40)	626	$ 4,703.60 (4,259.40, 5,147.80)	$ 1,070.60 (351.00, 1,790.10)
Male	569	$ 7,736.80 (6,868.60, 8,605.00)	569	$ 5,174.30 (4,696.90, 5,651.70)	$ 2,562.50 (1,588.80, 3,536.20)

CI: confidence interval

Appendix Table 6. Total and COVID-19-attributable healthcare costs (2023 CAD) standardized to 10 d stratified by neighborhood income quintile

Neighborhood Income quintiles	N	Exposed mean (95% CI)	N	Unexposed mean (95% CI)	Net cost mean (95% CI)
**Prediagnosis phase (30 d before index date)**					
1st quintile (lowest)	38,713	$ 130.10 (121.70, 138.50)	39,149	$ 120.90 (113.60, 128.20)	$ 9.70 (−0.80, 20.20)
2nd quintile	34,286	$ 108.90 (101.60, 116.20)	34,641	$ 107.10 (100.30, 114.00)	$ 2.20 (−7.20, 11.70)
3rd quintile	34,363	$ 96.80 (90.00, 103.60)	34,716	$ 100.30 (93.50, 107.10)	$ −3.00 (−12.20, 6.10)
4th quintile	28,162	$ 94.50 (86.80, 102.10)	28,378	$ 97.90 (89.90, 105.90)	$ −3.00 (−13.30, 7.30)
5th quintile (highest)	22,669	$ 93.30 (85.20, 101.50)	22,856	$ 97.40 (87.10, 107.80)	$ −3.80 (−16.50, 8.80)
**Acute phase (80 d including index)**					
1st quintile (lowest)	38,601	$ 407.10 (386.10, 428.00)	39,126	$ 105.40 (100.20, 110.50)	$ 301.80 (280.70, 322.90)
2nd quintile	34,195	$ 348.50 (328.00, 369.10)	34,618	$ 99.00 (93.60, 104.40)	$ 249.70 (228.80, 270.50)
3rd quintile	34,287	$ 304.30 (286.10, 322.60)	34,696	$ 92.40 (86.60, 98.10)	$ 212.10 (193.30, 230.80)
4th quintile	28,107	$ 292.80 (273.00, 312.60)	28,367	$ 83.60 (78.20, 89.00)	$ 209.30 (189.20, 229.40)
5th quintile (highest)	22,615	$ 284.70 (263.20, 306.10)	22,850	$ 83.80 (78.60, 89.00)	$ 201.00 (179.40, 222.60)
**Post-acute phase (81 to 360 d post index or to the start of the terminal phase, whichever occurred first)**					
1st quintile (lowest)	38,529	$ 132.50 (125.80, 139.10)	39,069	$ 109.30 (104.50, 114.10)	$ 23.70 (15.90, 31.50)
2nd quintile	34,125	$ 121.10 (112.80, 129.40)	34,569	$ 98.90 (94.00, 103.90)	$ 22.40 (13.20, 31.70)
3rd quintile	34,232	$ 102.80 (97.20, 108.40)	34,646	$ 89.20 (84.80, 93.70)	$ 14.00 (7.20, 20.70)
4th quintile	28,073	$ 95.80 (90.50, 101.00)	28,324	$ 84.30 (79.90, 88.80)	$ 11.80 (5.40, 18.30)
5th quintile (highest)	22,577	$ 98.50 (91.90, 105.20)	22,829	$ 87.00 (82.10, 92.00)	$ 11.80 (4.00, 19.60)
**Terminal phase, early deaths (deaths within 60 d of index)**					
1st quintile (lowest)	556	$ 8,988.90 (8,229.10, 9,748.70)	556	$ 5,022.70 (4,524.70, 5,520.70)	$ 3,966.20 (3,068.90, 4,863.40)
2nd quintile	454	$ 9,046.00 (8,253.10, 9,839.00)	454	$ 5,012.90 (4,465.70, 5,560.10)	$ 4,033.10 (3,123.90, 4,942.40)
3rd quintile	403	$ 8,320.50 (7,439.60, 9,201.40)	403	$ 4,284.20 (3,811.20, 4,757.20)	$ 4,036.30 (3,045.50, 5,027.20)
4th quintile	268	$ 8,480.20 (7,465.30, 9,495.00)	268	$ 4,918.60 (4,373.20, 5,464.00)	$ 3,561.50 (2,422.90, 4,700.20)
5th quintile (highest)	238	$ 8,445.70 (7,327.60, 9,563.80)	238	$ 4,582.10 (3,902.30, 5,261.90)	$ 3,863.60 (2,570.70, 5,156.50)
**Terminal phase, late deaths (deaths after 60 d of index)**					
1st quintile (lowest)	333	$ 6,854.10 (5,896.50, 7,811.70)	333	$ 5,506.80 (4,733.00, 6,280.60)	$ 1,347.30 (168.20, 2,526.50)
2nd quintile	284	$ 7,408.40 (6,238.40, 8,578.40)	284	$ 4,435.70 (3,930.10, 4,941.30)	$ 2,972.70 (1,740.10, 4,205.30)
3rd quintile	228	$ 6,636.20 (5,397.50, 7,875.00)	228	$ 5,196.80 (4,429.00, 5,964.60)	$ 1,439.40 (0.30, 2,878.50)
4th quintile	167	$ 6,133.00 (4,963.60, 7,302.50)	167	$ 4,757.70 (3,949.40, 5,566.00)	$ 1,375.30 (−52.40, 2,803.00)
5th quintile (highest)	183	$ 5,973.50 (4,728.30, 7,218.70)	183	$ 4,457.40 (3,771.70, 5,143.00)	$ 1,516.20 (79.50, 2,952.80)

CI: confidence interval

Appendix Table 7. Total and COVID-19-attributable healthcare costs (2023 CAD) standardized to 10 d stratified by resource utilization band

RUB	N	Exposed mean (95% CI)	N	Unexposed mean (95% CI)	Net cost mean (95% CI)
**Prediagnosis phase (30 d before index date)**					
Non-users	12,270	$ 3.20 (2.90, 3.50)	12,292	$ 3.40 (3.20, 3.60)	$ −0.20 (−0.60, 0.20)
Healthy Users	8,130	$ 7.40 (7.00, 7.80)	8,138	$ 8.50 (7.90, 9.10)	$ −1.10 (−1.80, −0.40)
Low resource utilization	28,086	$ 15.70 (14.90, 16.60)	28,131	$ 17.60 (16.60, 18.70)	$ −1.90 (−3.30, −0.50)
Moderate resource utilization	77,704	$ 53.60 (51.70, 55.50)	78,141	$ 57.80 (55.80, 59.80)	$ −4.20 (−6.90, −1.50)
High resource utilization	23,664	$ 176.40 (167.40, 185.40)	24,023	$ 189.10 (179.50, 198.60)	$ −12.60 (−25.50, 0.20)
Very high resource utilization	8,339	$ 958.60 (903.80, 1,013.40)	9,015	$ 805.90 (755.80, 856.00)	$ 153.30 (80.30, 226.30)
**Acute phase (79 d including index)**					
Non-users	12,268	$ 110.80 (88.70, 132.90)	12,291	$ 6.90 (6.10, 7.60)	$ 103.90 (81.80, 126.10)
Healthy Users	8,127	$ 76.80 (54.40, 99.20)	8,137	$ 12.10 (11.00, 13.20)	$ 64.70 (42.30, 87.10)
Low resource utilization	28,068	$ 112.60 (99.50, 125.70)	28,131	$ 20.70 (19.50, 22.00)	$ 91.90 (78.70, 105.10)
Moderate resource utilization	77,571	$ 276.00 (263.70, 288.30)	78,131	$ 59.30 (57.30, 61.20)	$ 216.70 (204.30, 229.20)
High resource utilization	23,581	$ 486.30 (461.70, 511.00)	24,012	$ 166.40 (158.60, 174.10)	$ 320.00 (294.40, 345.60)
Very high resource utilization	8,190	$ 1,796.10 (1,708.80, 1,883.40)	8,955	$ 630.70 (598.70, 662.80)	$ 1,165.70 (1,073.90, 1,257.50)
**Post-acute phase (81 to 360 d post index or to the start of the terminal phase, whichever occurred first)**					
Non-users	12,264	$ 23.60 (19.90, 27.30)	12,291	$ 13.40 (11.20, 15.60)	$ 10.20 (5.90, 14.50)
Healthy Users	8,126	$ 21.70 (15.40, 28.10)	8,134	$ 17.70 (14.40, 21.10)	$ 4.00 (−3.20, 11.20)
Low resource utilization	28,062	$ 30.40 (28.10, 32.60)	28,130	$ 26.40 (24.80, 27.90)	$ 4.00 (1.30, 6.70)
Moderate resource utilization	77,515	$ 79.60 (76.40, 82.80)	78,092	$ 66.10 (64.10, 68.10)	$ 13.50 (9.80, 17.20)
High resource utilization	23,530	$ 178.40 (170.10, 186.60)	23,966	$ 159.60 (153.10, 166.10)	$ 18.90 (8.50, 29.20)
Very high resource utilization	8,039	$ 743.80 (703.50, 784.10)	8,824	$ 580.10 (553.20, 607.10)	$ 164.40 (117.10, 211.60)
**Terminal phase, early deaths (deaths within 60 d of index)**					
Non-users	18	$ 9,762.40 (4,637.90, 14,887.00)	18	$ 6,109.10 (1,262.40, 10,955.80)	$ 3,653.30 (−2,943.70, 10,250.30)
Healthy Users	7	$ 13,280.20 (3,054.30, 23,506.20)	7	$ 2,008.90 (−452.10, 4,469.90)	$ 11,271.40 (3,288.30, 19,254.40)
Low resource utilization	56	$ 11,253.20 (8,758.80, 13,747.60)	56	$ 3,759.90 (2,482.80, 5,037.00)	$ 7,493.30 (4,700.90, 10,285.70)
Moderate resource utilization	553	$ 9,422.80 (8,582.60, 10,262.90)	553	$ 4,607.40 (4,151.30, 5,063.50)	$ 4,815.40 (3,898.50, 5,732.30)
High resource utilization	427	$ 8,589.40 (7,731.40, 9,447.40)	427	$ 4,444.80 (3,942.60, 4,947.10)	$ 4,144.50 (3,176.50, 5,112.60)
Very high resource utilization	858	$ 8,115.70 (7,600.70, 8,630.70)	858	$ 5,155.40 (4,786.50, 5,524.20)	$ 2,960.40 (2,334.80, 3,585.90)
**Terminal phase, late deaths (deaths after 60 d of index)**					
Non-users	17	$ 9,953.60 (3,943.20, 15,963.90)	17	$ 2,194.80 (529.40, 3,860.10)	$ 7,758.80 (2,175.00, 13,342.60)
Healthy Users	< = 5	$ 6,862.00 (−11,617.90, 25,341.90)	< = 5	$ 4,340.00 (−6,166.90, 14,846.90)	$ 2,522.00 (−8,245.50, 13,289.40)
Low resource utilization	23	$ 5,543.40 (2,198.10, 8,888.60)	23	$ 3,596.30 (1,429.20, 5,763.40)	$ 1,947.00 (−1,987.40, 5,881.50)
Moderate resource utilization	279	$ 7,754.90 (6,278.30, 9,231.40)	279	$ 4,928.70 (4,202.00, 5,655.30)	$ 2,826.20 (1,201.40, 4,451.00)
High resource utilization	231	$ 6,276.20 (5,133.40, 7,418.90)	231	$ 4,040.00 (3,434.50, 4,645.50)	$ 2,236.10 (947.30, 3,524.90)
Very high resource utilization	642	$ 6,364.70 (5,812.20, 6,917.30)	642	$ 5,369.50 (4,911.80, 5,827.30)	$ 995.20 (307.70, 1,682.60)

CI: confidence interval; RUB: resource utilization band

Appendix Table 8. Total and COVID-19-attributable healthcare costs (2023 CAD) standardized to 10 d after varying pre-diagnosis, acute and post-acute phase lengths

Costs categories	Exposed mean (95% CI) N = 159,817	Unexposed mean (95% CI) N = 159,817	Net cost mean (95% CI) N = 159,817
**Prediagnosis phase (120 d before index date)**			
Total costs	$ 102.70 (100.00, 105.40)	$ 97.20 (94.80, 99.60)	$ 5.50 (2.20, 8.90)
Costs by resource category			
Inpatient care	$ 25.00 (23.40, 26.70)	$ 23.90 (22.30, 25.40)	$ 1.20 (−1.00, 3.30)
Outpatient services	$ 16.90 (16.20, 17.70)	$ 14.60 (14.10, 15.20)	$ 2.30 (1.40, 3.20)
ED visits	$ 5.00 (4.90, 5.10)	$ 3.70 (3.60, 3.80)	$ 1.30 (1.20, 1.40)
Medications	$ 12.20 (11.80, 12.70)	$ 11.40 (10.90, 11.90)	$ 0.90 (0.20, 1.50)
Physician services	$ 4.90 (4.80, 5.00)	$ 5.20 (5.10, 5.20)	$ −0.30 (−0.30, −0.20)
Rehabilitation	$ 2.10 (1.70, 2.50)	$ 2.00 (1.70, 2.30)	$ 0.10 (−0.40, 0.60)
Complex care	$ 3.50 (2.80, 4.20)	$ 2.80 (2.20, 3.40)	$ 0.70 (−0.20, 1.60)
Homecare	$ 8.80 (8.30, 9.30)	$ 6.90 (6.60, 7.30)	$ 1.90 (1.30, 2.50)
Long term care	$ 0.00 (.,.)	$ 0.00 (.,.)	$ 0.00 (.,.)
Other	$ 1.90 (1.80, 1.90)	$ 2.80 (2.80, 2.80)	$ −0.90 (−1.00, −0.90)
**Acute phase (29 d including index)**			
Total costs	$ 556.10 (542.20, 570.00)	$ 99.70 (96.70, 102.70)	$ 456.50 (442.50, 470.50)
Costs by resource category			
Inpatient care	$ 378.90 (366.80, 391.00)	$ 21.80 (19.90, 23.70)	$ 357.10 (344.90, 369.30)
Outpatient services	$ 19.40 (18.60, 20.10)	$ 14.90 (14.20, 15.60)	$ 4.50 (3.50, 5.50)
ED visits	$ 39.90 (39.40, 40.50)	$ 3.70 (3.60, 3.80)	$ 36.20 (35.60, 36.80)
Medications	$ 10.90 (10.40, 11.40)	$ 11.90 (11.30, 12.50)	$ −1.00 (−1.80, −0.20)
Physician services	$ 13.90 (13.70, 14.00)	$ 7.50 (7.40, 7.50)	$ 6.40 (6.20, 6.60)
Rehabilitation	$ 6.20 (5.10, 7.20)	$ 1.90 (1.40, 2.50)	$ 4.20 (3.10, 5.40)
Complex care	$ 4.60 (3.70, 5.40)	$ 3.40 (2.70, 4.20)	$ 1.20 (0.10, 2.30)
Homecare	$ 11.10 (10.60, 11.70)	$ 8.10 (7.60, 8.50)	$ 3.20 (2.50, 3.80)
Long term care	$ 0.10 (0.00, 0.10)	$ 0.20 (0.10, 0.30)	$ −0.20 (−0.30, 0.00)
Other	$ 1.10 (1.10, 1.10)	$ 2.70 (2.70, 2.80)	$ −1.60 (−1.70, −1.60)
**Post-acute phase (81 to 360 d post index or to the start of the terminal phase, whichever occurred first)**			
Total costs	$ 130.70 (126.80, 134.60)	$ 95.60 (93.40, 97.70)	$ 35.40 (31.10, 39.70)
Costs by resource category			
Inpatient care	$ 44.10 (41.10, 47.10)	$ 22.30 (21.00, 23.60)	$ 21.90 (18.60, 25.10)
Outpatient services	$ 18.20 (17.50, 18.90)	$ 14.90 (14.40, 15.50)	$ 3.30 (2.40, 4.20)
ED visits	$ 5.00 (4.90, 5.10)	$ 3.60 (3.50, 3.60)	$ 1.40 (1.30, 1.60)
Medications	$ 12.10 (11.70, 12.50)	$ 11.80 (11.30, 12.20)	$ 0.30 (−0.30, 0.90)
Physician services	$ 4.60 (4.50, 4.60)	$ 4.60 (4.60, 4.70)	$ −0.10 (−0.10, 0.00)
Rehabilitation	$ 2.90 (2.60, 3.30)	$ 1.40 (1.20, 1.70)	$ 1.50 (1.10, 1.90)
Complex care	$ 5.30 (4.60, 6.00)	$ 3.00 (2.50, 3.60)	$ 2.30 (1.40, 3.20)
Homecare	$ 8.50 (8.10, 9.00)	$ 7.10 (6.70, 7.40)	$ 1.50 (1.00, 2.10)
Long term care	$ 4.00 (3.50, 4.40)	$ 2.70 (2.40, 3.10)	$ 1.20 (0.70, 1.80)
Other	$ 2.00 (2.00, 2.00)	$ 2.50 (2.50, 2.60)	$ −0.50 (−0.60, −0.50)

ED: emergency department; CI: confidence interval

Appendix Table 9. Total and COVID-19-attributable healthcare costs (2023 CAD), stratified by hospitalization and mortality

Total Cost during overall	Matched Exposed, N	Matched Exposed, 10d [a] mean (95%CI) costs	Attributable Costs 10d [a] mean (95%CI)	Matched Exposed, Total [b] mean (95%CI) costs	Attributable Costs Total [b] mean (95%CI)
No hospital stay within 14 d of index	151,716	$ 119 (114; 124)	$ 26 (20; 31)	$ 3,282 (3,211; 3,353)	$ 295 (207; 383)
Hospital stay within 14 d of index	8,101	$ 6,905 (6,627; 7,183)	$ 6,499 (6,221; 6,777)	$ 61,384 (59,589; 63179)	$ 50,490 (48,608; 52,372)
**Of those with a hospitalization within 14 d**					
No ICU stay	5,950	$ 3,610 (3,436; 3,784)	$ 3,174 (3,000; 3,348)	$ 41,522 (40,280; 42764)	$ 30,147 (28,760; 31,534)
ICU stay within 14 d of admission	2,126	$ 15,960 (15,140; 16,780)	$ 15,636 (14,818; 16,454)	$ 115,271 (110,181; 120,361)	$ 105,677 (100,467; 110,888)
ICU stay after 14 d of admission	25	$ 21,163 (13,537; 28,788)	$ 20,935 (13,902; 27,969)	$ 205,842 (108,022; 303,661)	$ 198,971 (107,437; 290,506)
**Of those with a hospitalization within 14 d and survival status**					
No ICU stay, alive at end of follow up	4,757	$ 1,083 (1,047; 1,119)	$ 746 (696; 796)	$ 38,987 (37,707; 40,267)	$ 30,030 (28,672; 31,389)
No ICU stay, died before end of follow up	1,193	$ 13,687 (13,118; 14,256)	$ 12,854 (12,275; 13,433)	$ 51,631 (48,180; 55083)	$ 30,610 (26,310; 34,911)

Total Cost during overall	Matched Exposed, N	Matched Exposed, 10d [a] mean (95%CI) costs	Attributable Costs 10d [a] mean (95%CI)	Matched Exposed, Total [b] mean (95%CI) costs	Attributable Costs Total [b] mean (95%CI)
CU stay within 14 d of dmission, alive at end of ollow up	1,300	$ 3,410 (3,221; 3,598)	$ 3,164 (2971; 3,357)	$ 122,744 (115,957; 129,532)	$ 114,945 (108,101; 121,790)
CU stay within 14 d of dmission, died before end of ollow up	826	$ 35,712 (34,529; 36,894)	$ 35,266 (34,078; 36,453)	$ 103,509 (95,976; 111,042)	$ 91,091 (83,205; 98,977)
CU stay after 14 d of dmission, alive at end of ollow up	10	$ 6,231 (2,270; 10,193)	$ 6,160 (2,883; 9,437)	$ 224,325 (81,710; 366,940)	$ 221,754 (103,788; 339,720)
CU stay after 14 d of dmission, died before end of ollow up	15	$ 31,117 (21,527; 40,707)	$ 30,786 (22,388; 39,183)	$ 193,519 (46,675; 340,364)	$ 183,783 (53,625; 313,941)

CAD: Canadian dollar, CI: confidence interval; d: day; ICU: intensive care unit

[a] Mean 10 d costs, rounded to the nearest dollar, for the period from index to end of follow-up or death whichever occurred first.

[b] Mean total costs, rounded to the nearest dollar, from index to end of follow-up or death whichever occurred first.

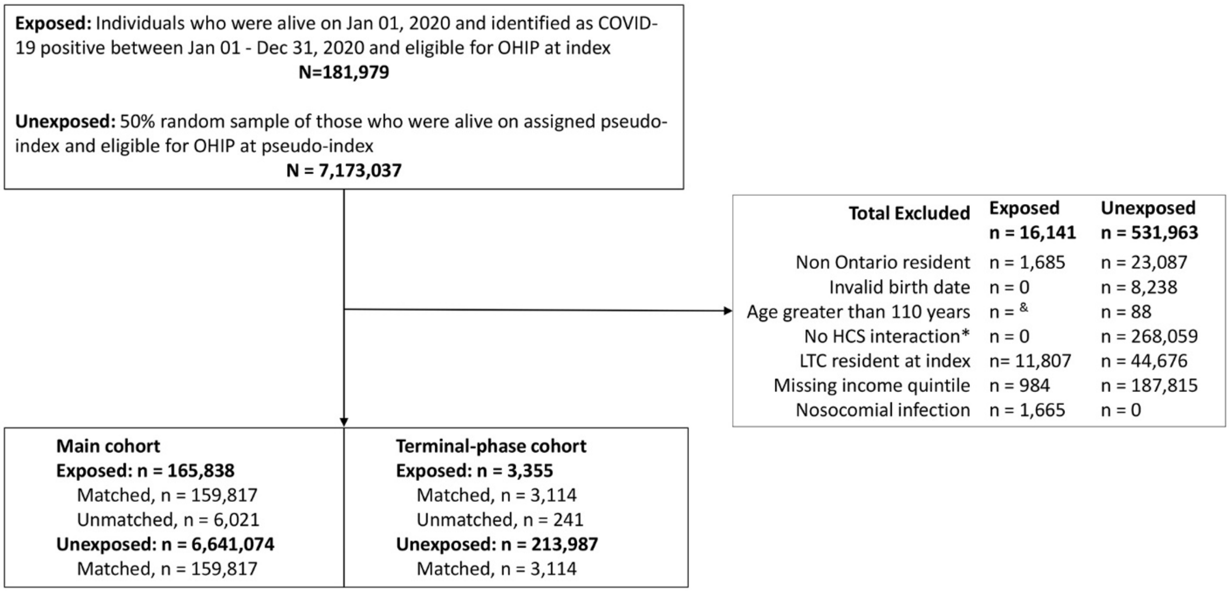

Appendix Figure. Cohort creation flowchart. HCS: healthcare system interaction; LTC: long-term care. [&]Due to small cell number this category has been combined with the "Non Ontario resident" category. *No interaction with healthcare system was defined as being aged ≥65 years without healthcare system interaction for the three years prior to the study start date or being <65 years without healthcare system interaction for the 10 years prior to the study start date
